# An economic model to evaluate cost-effectiveness of computer assisted knee replacement surgery in Norway

**DOI:** 10.1186/1471-2474-14-202

**Published:** 2013-07-06

**Authors:** Øystein Gøthesen, James Slover, Leif Havelin, Jan Erik Askildsen, Henrik Malchau, Ove Furnes

**Affiliations:** 1 Department of Orthopaedic Surgery, Haugesund Hospital, Karmsundsgate 120, 5528, Haugesund, Norway; 2 Department of Orthopaedic Surgery, New York University (NYU) Hospital for Joint Diseases, 301 East 17th Street; Suite 1616, New York, NY 10003, USA; 3 The Norwegian Arthroplasty Register, Department of Orthopedic Surgery, Haukeland University Hospital, Jonas Lies vei 65, 5053, Bergen, Norway; 4 Department of Surgical Sciences, University of Bergen, 5021, Bergen,Norway; 5 Stein Rokkan Centre for Social Studies, Uni Research, University of Bergen, 5021, Bergen, Norway; 6 Department of Orthopaedic Surgery, Massachusetts General Hospital, 55 Fruit Street, Boston, MA 02114, USA

**Keywords:** Artrhroplasty, Computer navigation, Cost-effectiveness, Health economy, Register, Markov

## Abstract

**Background:**

The use of Computer Assisted Surgery (CAS) for knee replacements is intended to improve the alignment of knee prostheses in order to reduce the number of revision operations. Is the cost effectiveness of computer assisted surgery influenced by patient volume and age?

**Methods:**

By employing a Markov model, we analysed the cost effectiveness of computer assisted surgery versus conventional arthroplasty with respect to implant survival and operation volume in two theoretical Norwegian age cohorts. We obtained mortality and hospital cost data over a 20-year period from Norwegian registers. We presumed that the cost of an intervention would need to be below NOK 500,000 per QALY (Quality Adjusted Life Year) gained, to be considered cost effective.

**Results:**

The added cost of computer assisted surgery, provided this has no impact on implant survival, is NOK 1037 and NOK 1414 respectively for 60 and 75-year-olds per quality-adjusted life year at a volume of 25 prostheses per year, and NOK 128 and NOK 175 respectively at a volume of 250 prostheses per year. Sensitivity analyses showed that the 10-year implant survival in cohort 1 needs to rise from 89.8% to 90.6% at 25 prostheses per year, and from 89.8 to 89.9% at 250 prostheses per year for computer assisted surgery to be considered cost effective. In cohort 2, the required improvement is a rise from 95.1% to 95.4% at 25 prostheses per year, and from 95.10% to 95.14% at 250 prostheses per year.

**Conclusions:**

The cost of using computer navigation for total knee replacements may be acceptable for 60-year-old as well as 75-year-old patients if the technique increases the implant survival rate just marginally, and the department has a high operation volume. A low volume department might not achieve cost-effectiveness unless computer navigation has a more significant impact on implant survival, thus may defer the investments until such data are available.

## Background

**Figure 1:**
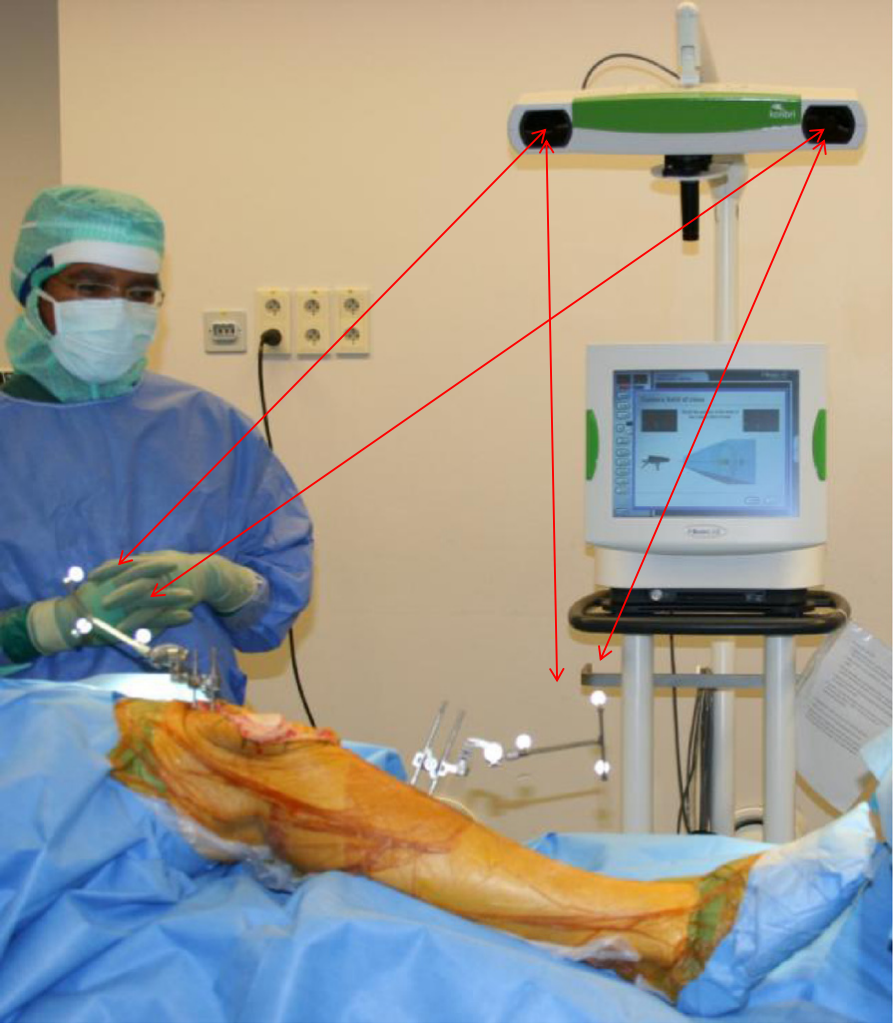
**Infrared rays are reflected from reflection balls attached to the tibia and femur and back to the camera and the computer. **The reciprocal distances and movements measured between the balls are registered by the computer which builds a model of the extremeties axes and anatomy. Surgical instruments are navigated according to the same principle.

Total knee replacement is considered a cost effective surgical procedure of considerable benefit to the patient. Patients experience a markedly improved quality of life after this type of intervention [1]. On the other hand, there is a risk that aseptic loosening, malalignment and instability, patellar pain or infection, may lead to poorer functionality and quality of life for the patient [2,3]. Over the last decade computer assisted orthopaedic surgery has undergone development, and the use of this type of navigation system is becoming increasingly common (Figure 1). In 2008, 19% of all primary knee replacements in Norway were computer assisted [4]. Better positioning of the prosthesis will in theory reduce the number of revisions [5,6].

A number of randomised studies have demonstrated better positioning of components when computer navigation has been used [7]. The follow-up time for these studies is short, and the results vary when it comes to improved functionality [8]. So far, no-one has been able to demonstrate that computer assisted surgery reduces the number of revision operations. Computer navigation equipment is expensive, and its use prolongs the operation time [7]. Hospitals have scarce resources at their disposal and consequently it is important that the cost effectiveness of new methods and new technology is evaluated, to ensure that every penny is spent on achieving optimal health effects. Within the field of knee replacement surgery good instrumentation is already in use, which is why we need to be extra critical whenever new methods are introduced. History has taught us that new technology and new methods are best introduced in stages, before the market is let loose. This approach provides an opportunity to discover weaknesses at an early stage, to prevent unnecessary harm to patients and the waste of public funds [9]. The Boneloc cement case (used to fix prostheses) which involved 20 Norwegian hospitals from 1991–1993, is but one example demonstrating the importance of thorough evaluation and testing [10]. In theory, computer assisted surgery should result in a better quality of life for the patient, measured in quality-adjusted life years, by reducing the probability of revisions. This model is supposed to guide health care providers in their investments and implementation of new technology. When considering an investment in CAS, it is important to have an idea of what impact this new technology is required to have on patient outcome, in order to achieve cost-efficiency for different age groups and hospital sizes (patient volumes). From the point of view of a healthcare enterprise, we wish to compare the cost per quality-adjusted life year gained by using computer navigation and conventional total knee arthroplasty (TKA) respectively. We also wish to discover how age, patient volume and revision probability influence the cost effectiveness.

## Methods

### Economic evaluation

The relative profitability of two alternative technologies, computer assisted and convensional surgery, is established using a cost effectiveness analysis. This type of comparison needs to consider possible changes to both benefits and costs. New technology may be cheaper or more expensive, and may have a better or worse impact compared to traditional technology. If computer assisted surgery proves to be cheaper and better, or poorer and more expensive, the solution is trivial, since one technology is dominant. The need for deliberation arises if both costs and benefits change in the same direction. This is normally presented in the form of an incremental cost-effectiveness ratio – ICER, i.e. an equation showing the change in cost relative to the change in effect for the two alternatives. This provides a cost per unit of benefit gained, which in turn may be compared to society's demand for useful employment of resources. In Norway, common practice uses a threshold value of NOK 500,000 for acceptable cost per quality-adjusted life year gained [11]. This does not mean that every intervention that scores below the threshold value should necessarily be accepted. It is also necessary to consider the intervention in relation to the resources available. Consequently, it is important to clarify the perspective of the analysis - patient, healthcare enterprise or society. Our analysis considers the benefits and costs from the point of view of a healthcare enterprise, while more indirect social costs, to relatives for instance, or the cost of absence from work, are excluded.

The measure of benefit is a quality-adjusted life year. This means that consideration is given not only to survival, but also to the quality of the patient’s health, measured on a scale from 0 (dead) to 1 (in perfect health), and for how long this health state lasts. There are a number of methods for measuring quality of life. Based on hypothetical questions about what one is willing to sacrifice in order to go from a poor state of health to a perfect state of health, along a number of different dimensions of weakened health, it is possible to arrive at a utility value. The utility values used here have been calculated by means of EQ-5D, a standardised questionnaire (developed by the EuroQol Group) which includes the five dimensions of mobility, self-care, usual activities, pain/discomfort, and anxiety/depression. Each dimension has three levels – no problems, some problems, extreme problems. By establishing the number of years during which patients experience the different utility values, we arrive at quality-adjusted life years. In turn these can be summarised for a patient population, in order to find the total benefit levels (measure of benefit) to be compared against the costs.

A treatment outcome is often uncertain, and a number of possible states may be envisaged. This means that the costs and benefits included are uncertain values, and we need to take account of the different treatment outcomes by adjusting for this uncertainty. By using a Markov decision model we are able to draw up a useful and clear presentation of different outcomes and their associated probabilities.

### Model

**Figure 2:**
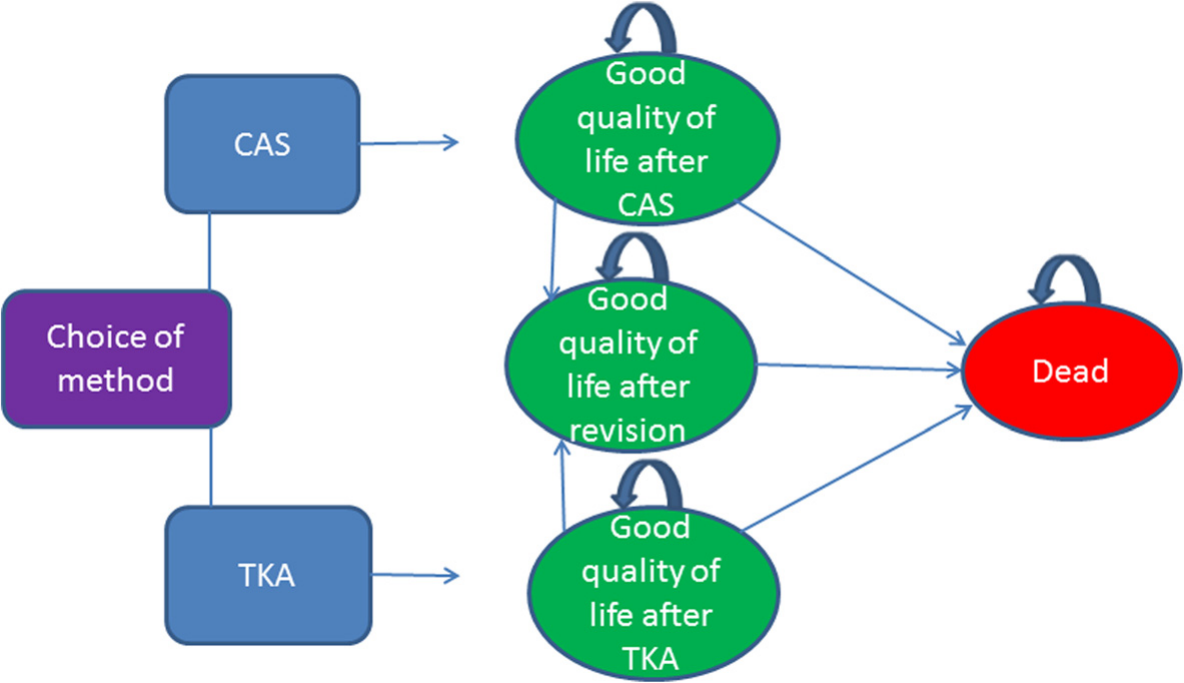
**The Markov Model. **The patient undergoes a total knee replacement operation, either by computer assisted surgery (CAS) or conventional total knee arthroplasty (TKA). If the patient survives the operation, he remains in perfect health until he dies of other causes, or needs a revision. The model comprises 20 yearly cycles until all patients have reached the health state of “dead”. In each cycle, the patients can either retain the same health state or go to a different health state. The benefits of each surgical method are measured in quality-adjusted life years (QALYs) for each cycle and are summarised after 20 cycles.

A Markov decision model is used to analyse various matters in a number of cycles (20 years in this model). In our model, a cycle equalled one year. We looked at the probability of certain occurences, such as revision and death, within each cycle. Since each occurence had an associated probability, this probability could be used to calculate the relevant costs and utility values within the same cycle (Figure 2).

Costs and utility values were allocated to each primary procedure and revision procedure. In this model, the patients went from one health state to another at an age-specific frequency and probability based on Norwegian data sources. The theoretical patient cohort accumulated costs and utility values over time. All costs and utility values accumulated over zero time were discounted at 4% per year [12]. The impact of alternative assumptions about the discount rate was tested using sensitivity analyses. Based on the Markov model, we deduced total costs and quality-adjusted life years to evaluate the cost effectiveness of conventional surgical techniques and computer assisted surgery. The model was constructed using a decision analysis software (TreeAge Pro 2009, Williamstown, MA).

The model was based on the following premise: 1) Patients who have their total knee prosthesis implanted by conventional surgery or by computer assisted surgery demonstrate the same post-operative utility value. 2) Mortality after the first year is the same as for other patients the same age who have not undergone this type of operation. 3) In this model, the patients will need only a single revision operation, and they have utility values allocated for the rest of their lives that match the value normally achieved following a single revision. The values of the various model parameters are given in Table 1.

**Table 1:** Model parameters and premise

	**TKA**	**CAS**	**Revision**
**Parameter values obtained from the literature**			
Utility values			
Postoperative	0.73 ^(30)^	0.73 ^(30)^	0.6 ^(3)^
Preoperative	0.4 ^(30)^	0.4 ^(30)^	0.73
**Estimated/obtained parameter values**			
Disutility value	-0.1	-0.1	-0.2
Mortality, 1st year after operation	0,63%	0,63%	
Mortality, remaining lifetime	Table B	Table B	
	Additional file	Additional file	
Cost (NOK) based on DRG	146.135^(28)^	146.135^(28)^	192.418^(28)^
Implant survival /probability of revision	Table A	Table A	
	Additional file	Additional file	
Number of revisions	1	1	No re-revision
Annual added cost* of computer navigation (NOK)			
Alternative 1		216.500	
Alternative 2		433.000	

*Total added cost for computer navigation equipment, software and maintenance contract per year. The cost per patient is described in the method section and depends on patient volume.

### Cohorts

We have undertaken an analysis of two groups of patients: 60-year-olds and 75-year-olds.

### Implant survival

Stipulations were made for implant survival and yearly probability of revision within the two cohorts based on data from the Norwegian Arthroplasty Register for patients over and under the age of 70 who had undergone surgery without computer assistance. For the younger cohort (60-year-olds) the implant survival and probability of revision in the model were set equal to the data for patients under the age of 70, whereas implant survival and probability of revision for the older cohort (75-year-olds) were set equal to the data for patients over 70 years of age (Table D in Additional file 1). For years 1 to 11 we used register data to find the yearly probability of revision by means of the Kaplan-Meier method. For years 12 to 20 we estimated the probability of revision to match the results reported by the Swedish knee arthroplasty register and large-scale cohort studies [13-16]. We have used probability of revision (100% minus implant survival ) as a concept in the model, but since Norwegian practice traditionally gives implant survival (100-probability of revision), we calculate the corresponding 10-year implant survival by making an approximation that the probability of revision is the same from year to year (both values are given in Table A in the Additional file 1).

The Norwegian Arthroplasty Register was established in 1987 by the Norwegian Orthopaedic Association; it is publicly funded and is independent of the implant industry [17,18]. The register started collecting data for conventional total knee arthroplasty (TKA) in 1994 [2]. Norwegian surgeons have reported 99% of primary knee prostheses and 97% of revisions [19].

### Probability of death

The probability of death within the first year, including perioperative death, was set to 0.63% for cohort 1 and 2.40% for cohort 2, based on linked data from the Norwegian Arthroplasty Register and the National Register for 60 and 75-year-olds. Probability of death after the first year, irrespective of knee replacement surgery, was set equal to the age-specific mortality in the population [20] (Table B in Additional file 1). Studies have shown that the mortality rate is higher for knee replacement revision surgery than for primary knee replacement surgery [21]. The perioperative mortality was therefore set 50% higher for revisions within this model, to 0.95% and 3.60% respectively for cohorts 1 and 2.

### Utility values

Patients who receive total knee replacement surgery are expected to enjoy the same quality of life on completion of the postoperative phase and rehabilitation period whether their surgery was conventional or computer assisted. The utility values that were used in the model were based on findings from earlier publications on arthroplasty surgery [22,23]. The pre-operative value was set to 0.40, the post-operative value to 0.73 (the operation provides an improvement of 0.33). These values are similar to those found in the Swedish hip arthroplasty register, and match the values found for knee replacements [24]. The values are here based on EQ-5D, which is a commonly used instrument for measuring quality of life. Studies have shown that the results following revision replacement surgery is poorer than after primary replacements [3,22]. The value following knee replacement revision surgery was therefore set to an initial value of 0.60.

### Disutility value

The disutility value represents the disutility of the reduced quality of life experienced by the patient in connection with a particular health state or clinical outcome [25]. In this model, disutility values represent the reduced quality of life a patient might experience in connection with the operation. The disutility value includes any reduced mobility, increased pain and potential complications that the patient may experience in the perioperative phase. The value is given at the time a patient is undergoing a procedure in the model. The disutility values of conventional knee replacement surgery, computer assisted knee replacement and the revision prosthesis operation, were entered into the model and contributed to a downward adjustment of quality-adjusted life years compared to the patient’s total value of quality-adjusted life years. The disutility value of a total knee replacement was set to -0.1 on a discretionary basis and was only allocated to the first post-operative year (i.e. a utility value of 0.73-0.1=0.63 in the model’s first cycle). Some have pointed out the risk of increased perioperative morbidity in connection with computer assisted knee replacement operations due to the risk of fracture and infection associated with the positioning of external fixation pins in bone, as well as a longer operation time. However, the incidence of these complications is so low that we allocated the same disutility value to computer navigation as to the conventional technique [26,27]. Revisions, which involve a higher frequency of complications and a longer training period than primary knee replacements, were allocated a value of -0.2 quality-adjusted life years.

### Costs

The added cost of computer navigation includes expenditure such as computer hardware and knee replacement software, instruments and maintenance contracts. This was estimated at NOK 1,082,500 per department per year. Disposable equipment (reflection balls) constituted an additional cost, set to NOK 200 per operation. The costs are based on prices obtained from Brainlab Scandinavia, which is a frequently used supplier of computer navigation equipment but supplies no prostheses. The annual cost was calculated based on a five-year usage period for the equipment; the additional cost per department per year was then calculated to NOK 216,500. The cost of disposable equipment was additional. The annual cost was divided by the number of patients operated on at the hospital, in order to find the added cost per operation. Frequent upgrades and new technology may be envisaged to drive the costs up. Consequently, we also looked at the outcome in a scenario where prices were increased by 100%, i.e. to NOK 433,000. The cost per operation, without the use of computer navigation, was based on DRG rate 209A (NOK 146,135) for primary prostheses and 209B (NOK 192,418) for revision prostheses in 2011, which gives the average total cost of these operations at Norwegian hospitals [28]. We expected the hospitalisation periods and staff requirements to be equal with computer navigation and the traditional method.

### Analysis

The ICER (”incremental cost-effectiveness ratio”) was found by dividing the difference between total accumulated costs (including the cost of future knee replacement revisions) by the difference in total quality-adjusted life years gained for each of the surgical methods. As in accordance with the guidance provided by the UK National Institute for Clinical Excellence (NICE), our calculations did not include loss of productivity [29]. In other words, our strategy was to find parameter values based on today’s literature and data that would produce as true a picture as possible (Table 1). Each cycle (each year) of the model was analysed with respect to accumulated costs and quality-adjusted life years. Finally, the total cost and total number of quality-adjusted life years were analysed for each of the surgical methods (computer navigation and conventional arthroplasty) when all patients included in the model had reached the health state of dead. We used sensitivity analyses to test the stability of the conclusions by varying the parameter values above a certain interval, to see what effect they had on the outcome (ICER). A two-way sensitivity analysis was used for the two age cohorts in order to investigate the relationship between patient volume, the probability of revision, and the cost effectiveness of computer assisted surgery in Norway (Table D in Additional file 1).

### Ethics

The Norwegian Arthroplasty Register has permission from the Norwegian Data Inspectorate to collect patient data, based on obtaining written consent from patients (last issued May 24, 2004; reference number 2003/58-3).

## Results

In the course of 20 years (20 cycles in the Markov model) a 60-year-old is expected to gain 7.44 quality-adjusted life years, while a 75-year-old would gain 5.46 quality-adjusted life years. The cost of these quality-adjusted life years depends on the patient volume and whether any revision surgery is required. At the outset, we assumed that the probability of revision is identical for conventional atrhroplasty and computer assisted surgery. At a volume of 250 prostheses per year, the cost of conventional arthroplasty in 60-year-olds is NOK 340,606; with computer assisted surgery the cost is NOK 341,558. For 75-year-olds, the cost of conventional arthroplasty is NOK 335,994 while the cost of computer assisted surgery is NOK 336,946. For conventional arthroplasty, this amounts to a cost per quality-adjusted life year of NOK 45,762 for 60-year-olds; for computer assisted surgery, the corresponding figure is NOK 45,890, which is a difference of NOK 128. For 75-year-olds the cost per quality-adjusted life year undergoing conventional arthroplasty is NOK 61,537; for computer assisted surgery, the corresponding figure is NOK 61,712, which is a difference of NOK 175 per quality-adjusted life year. If we make a similar calculation for a volume of 25 prostheses per year, we find that the added cost of computer navigation amounts to NOK 1,037 per quality-adjusted life year for 60-year-olds and NOK 1,414 for 75-year-olds. These values represent a base case before we take account of changes to the probability of revision following the introduction of the new method and how the result is impacted by increased costs.

**Figure 3:**
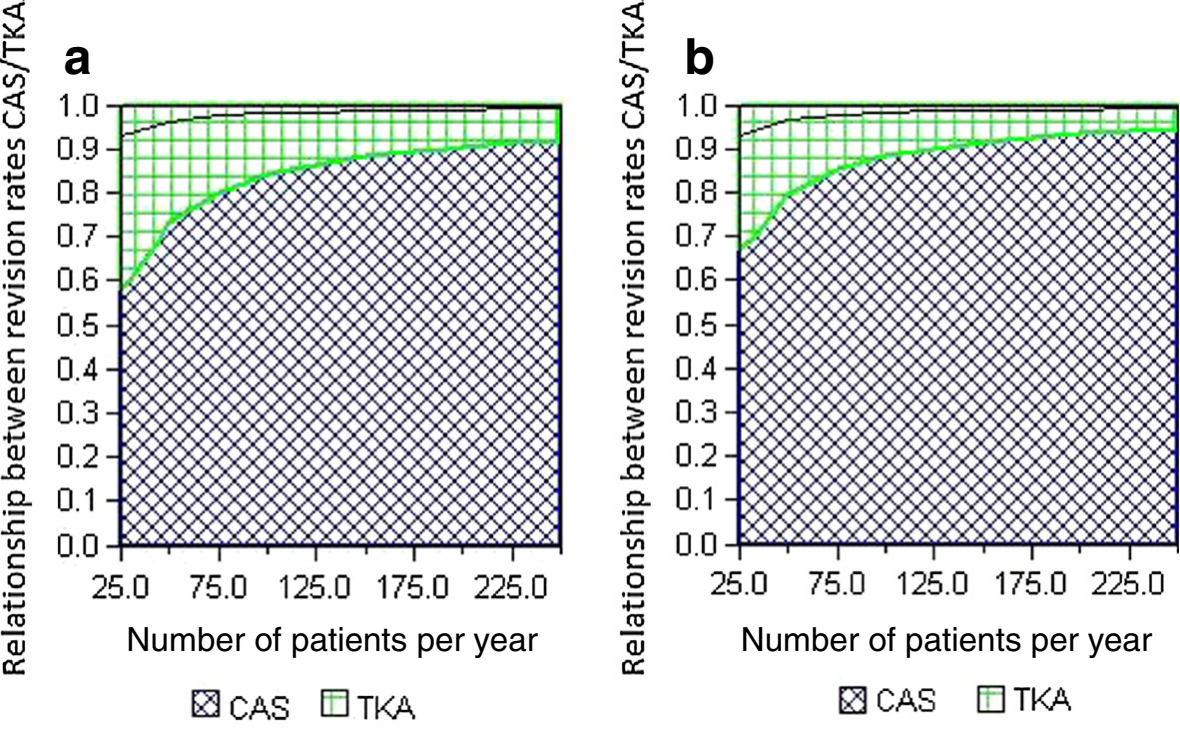
**The results of the sensitivity analysis for patient volumes in a) cohort 1 (age 60) and b) cohort 2 (age 75).** The blue cross-hatched areas show when computer navigation is cost effective. The area between the threshold (black line) and the blue cross-hatched area shows when the cost of computer navigation does not exceed the healthcare sector’s willingness to pay per QALY.

Figures 3a and b show that when the probabilities of revision are equal, the cost per quality-adjusted life year is higher for computer assisted knee replacement surgery than for conventional knee replacements, and there are no savings to be made. Should the probability of revision be improved, the number of quality-adjusted life years will increase and therefore reduce the cost per quality-adjusted life year. If the improvement is considerable, savings may be made. Given that the health care sector’s maximum threshold value for acceptable added cost (ICER) is NOK 500,000 per quality-adjusted life year gained, we find in both cohorts that a small improvement of implant survival is required to get below the threshold. At low patient volumes and a low impact on the probability of revision, we will risk surpassing the threshold value (Tables C and D in Additional file 1).

**Figure 4:**
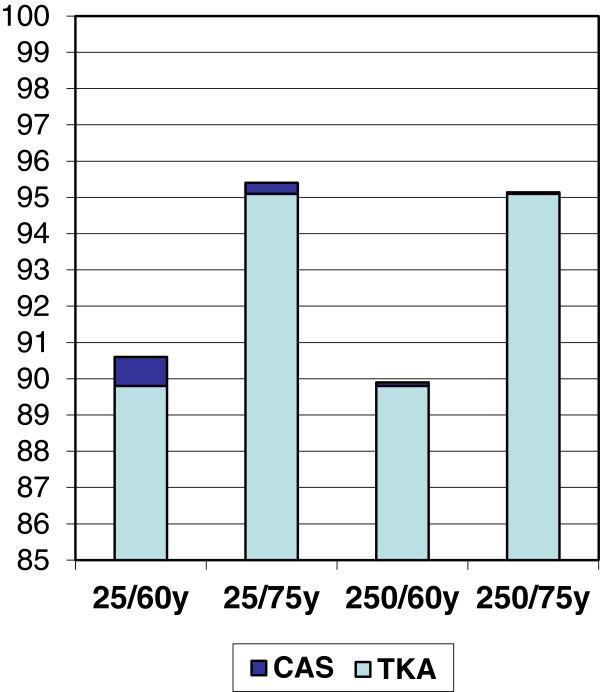
**The dark blue areas of the columns illustrate the improvement in 10-year Kaplan-Meier implant survival which is required for computer navigation not to exceed the healthcare sector’s NOK 500,000 threshold.** For example, the column to the far left (25/60 years of age) illustrates this for a hospital with a low patient volume (25 knee replacements per year) and a younger population (age 60).

In order to get below the threshold of the sector’s willingness to pay, the probability of revision will have to fall by at least 7.5% (of 10.2%) for cohort 1 at a volume of 25 knee replacements per year, and by at least 1% at a volume of 250 knee replacements per year. For cohort 2 the probability of revision needs to fall by at least 7% (of 4.9%) at a volume of 25 prostheses per year and by at least 1% at a volume of 250 prostheses per year. If we convert this information, we find that the improvement needs to increase the 10-year implant survival in cohort 1 from 89.8% to 90.6% at a volume of 25 prostheses per year, and from 89.8 to 89.9% at 250 prostheses per year. In cohort 2 implant survival needs to improve from 95.1% to 95.4% at a volume of 25 prostheses per year and from 95.10% to 95.14% at a volume of 250 prostheses per year (Figure 4). The probability of getting below the ICER threshold is virtually the same for the older cohort as for the younger cohort.

Doubling the cost had little impact on the probability of getting below the threshold value of NOK 500,000 at high patient volumes. For low patient volumes, doubling the cost would require further improvement of implant survival (for cohort 1: from 90.6% to 91.1% and for cohort 2: from 95.4% to 95.7%), to get below the healthcare sector’s threshold value of NOK 500,000 per quality-adjusted life year (Table C in Additional file 1: Figures 5a, 5b, 6a and 6b in Additional file 1).

A sensitivity analysis of variations between 1% and 10% to the discount rate showed no impact on the results.

## Discussion

The model suggests that computer navigation may be an alternative to today’s conventional total knee replacement, provided there is proven reduction in the probability of revision, and provided the price of navigation equipment does not rise. To date, no studies have documented that computer navigation causes such reduction. In order to get below the healthcare sector’s threshold value for cost added per quality-adjusted life year gained, the probability of revision needs to be reduced by somewhere between 0.8% and 13.0%, depending on patient volume and the cost of the computer navigation equipment. It is clear that patient volume, not surprisingly, impacts significantly on the cost effectiveness of computer navigation. At high patient volumes the improvement required is less than at low patient volumes. Age appears not to influence the probability of getting below the threshold value to any great extent, but there is a minor trend indicating that the probability is greatest in the older cohort, particularly at low patient volumes.

The information provided by this analysis is valuable to hospitals and health politicians focusing on areas that provide as much health as possible for the money. Moreover, the model may be transferred to other high-cost surgical procedures, particularly within areas covered by quality registers that are in a position to provide much valuable information. Ever-increasing healthcare costs make it increasingly important to evaluate the usefulness of new technology. Two of the authors recently published an analysis of the cost effectiveness of computer navigation and knee replacement surgery in the US [30]. They investigated the impact of patient volume on cost effectiveness. It was found that it would be more difficult to achieve cost effectiveness at low patient volumes than at high patient volumes. Norwegian circumstances are significantly different from American circumstances in a number of ways. Our costings are based on prices in the Norwegian market and to the Norwegian Health Service, which are different from those available in the USA. Also, the implant survival used in this analysis is based on figures obtained from the Norwegian Arthroplasty Register.

### Strengths and weaknesses

An important strength of this analysis is the use of implant survival data from the Norwegian Arthroplasty Register, which includes prospective data about more than 26,000 total knee replacements [31]. This data strengthens the analysis in that it allows for the probability of revision to be specified year by year, based on results reported by a number of different surgeons at different hospitals in a single country. By combining this data with cost and mortality data from the same country, the analysis becomes relevant at number of levels within the Norwegian Health Service. However, the register holds data only for the last 11 years (at the time of analysis), which meant we had to estimate the implant survival rate for all earlier years.

The model does not take account of the cost of increased operation time. We know that the operation time for bicompartmental knee prostheses in Norway has fallen from an average of 109 minutes in 1994 to 96 minutes in 2008. When using computer navigation, the operation time rose in the period 2005–2008 to 107 minutes in 2008, probably due to a rise in the spread of such navigation equipment combined with limited experience of its use [32]. For beginners, the procedure will be time-consuming, but given experience and technology improvements the operation time is likely to be considerably reduced. Furthermore, the cost of longer operation times will depend on the organisation’s ability to make alternative use of the time saved. The model may therefore over estimate or under estimate the real cost of the procedures.

Utility values are extrapolated in a number of different ways, which means there may be a number of different utility values for a given state [33]. We have looked at values within prosthesis surgery and compared two groups which at first appear identical. However, the values quoted in the literature differ considerably for the same states. There is a risk of over estimating or under estimating the values and this may impact on the result, but because we limit our analysis to arthroplasty and compare primary operations to revision operations, the consequence of any erroneous estimates will be kept to a minimum.

Another limitation of the analysis is the estimate of probability of changing health states. Data used to determine the yearly probability of revision include patients that had their prosthesis implanted many years ago, without allowing for later developments with respect to technique, material and design. The estimated probabilities may therefore differ from the real values for today’s knee replacement patients in Norway, and also may differ between countries. The threshold to perform a revision may be affected by socio-economical state, patient co-morbidities and surgeon’s experience, which may differ between countries and regions Furthermore, the analysis does not take account of re-revisions. The probability of re-revisions for reasons of aseptic loosening or prosthesis infection may not be the same in both groups, and this may have impacted on the result of the analysis. The frequency of complications such as thromboembolism, infection and postoperative confusion, may also be different. Furthermore, earlier studies based on Norwegian register data have indicated an increased risk of aseptic loosening and infection with longer operation times [34]. If computer navigation leads to longer operation times, this may impact negatively on the outcomes for this procedure.

We found that high volume centres are more likely to achieve cost-effectiveness. On the other hand, small volume centres might imply that the knee surgeons have a low volume and thereby less experience. Thus, the need of a more precise technology might be greater in a small volume centre. This aspect must be evaluated when considering investments in this new technology.

Further studies, including register studies and randomised studies with long-term follow-ups, are necessary to prove any differences in outcomes between the two surgical techniques. In particular, any impact that computer navigation may have on implant survival will be crucial. It is of considerable concern that there may even be an increased risk of revision in the short term, when computer is being used [32].

## Conclusions

The healthcare sector’s willingness to pay may be expected to cover the added cost of computer assisted knee replacement surgery provided the patient volume is large and there is positive impact on implant survival. The probability of getting below the financial threshold for added cost per quality-adjusted life year gained, is falling at rate with falling patient volumes and falling survival rates. The patients’ age has little impact. The new technique should be carefully tested in a group of hospitals with different age groups and patient volume to evaluate the long term outcome. This model estimates required survival rates to achieve cost-effectiveness with CAS. Until such results are achieved and reported from clinical trials, we suggest deferral of extended investments in computer navigation technology.

## Competing interests

Conflicts of interest declared: The project’s main sponsor is the Research Council of Norway and Øystein Gøthesen has had a PhD grant.

## Authors' contributions

JS and ØG performed the statistical analysis and drafted the manuscript. OF, LH, JEA and HM participated in its design, coordination and interpretation of the results, and helped to draft the manuscript. All authors read and approved the final manuscript.

## Pre-publication history

The pre-publication history for this paper can be accessed here:

http://www.biomedcentral.com/1471-2474/14/202/prepub

## Supplementary Material

Additional file 1 Appendix. Click here for file

## References

[B1] Ethgen O Bruyere O Richy F Dardennes C Reginster JY Health-related quality of life in total hip and total knee arthroplasty. A qualitative and systematic review of the literature J Bone Joint Surg Am 2004 86-A 5 963 974 15118039 10.2106/00004623-200405000-00012

[B2] Furnes O Espehaug B Lie SA Vollset SE Engesaeter LB Havelin LI Early failures among 7,174 primary total knee replacements: a follow-up study from the Norwegian Arthroplasty Register 1994–2000 Acta Orthop Scand 2002 73 2 117 129 10.1080/000164702753671678 12079006

[B3] Saleh KJ Celebrezze M Kassim R Dykes DC Gioe TJ Callaghan JJ Functional outcome after revision hip arthroplasty: a metaanalysis Clin Orthop Relat Res 2003 416 254 264 14646768 10.1097/01.blo.0000093006.90435.43

[B4] Furnes O Havelin LI Espehaug B Steindal K Soeraas TE The Norwegian arthroplasty register Annual report 2009 2009 62

[B5] Jeffery RS Morris RW Denham RA Coronal alignment after total knee replacement J Bone Joint Surg Br 1991 73 5 709 714 1894655 10.1302/0301-620X.73B5.1894655

[B6] Ritter MA Faris PM Keating EM Meding JB Postoperative alignment of total knee replacement. Its effect on survival Clin Orthop Relat Res 1994 299 153 156 8119010

[B7] Bauwens K Matthes G Wich M Gebhard F Hanson B Ekkernkamp A Navigated total knee replacement. A meta-analysis J Bone Joint Surg Am 2007 89 2 261 269 10.2106/JBJS.F.00601 17272438

[B8] Longstaff LM Sloan K Stamp N Scaddan M Beaver R Good alignment after total knee arthroplasty leads to faster rehabilitation and better function J Arthroplasty 2009 24 4 570 578 10.1016/j.arth.2008.03.002 18534396

[B9] Graff BA Thurmer H Soreide O Norderhaug IN When new methods are about to be introduced Tidsskr Nor Legeforen 2008 14 128(4) 472 18274585

[B10] Furnes O Hip prostheses and cements Tidsskr Nor Legeforen 2004 23 124(18) 2395 15467812

[B11] Saelensminde K IS-1435: Helseeffekter i samfunnsøkonomiske analyser 2007 26 Published by Sosial- og helsedirektoratet, Helseøkonomi og finansiering, Pb. 7000 St Olavs plass, 0130 Oslo http://www.kvalitetogprioritering.no/saker/_attachment/12363?_download=true&_ts=118895adfb8

[B12] Veileder i samfunnsøkonomiske analyser 2005 35 Published by Finansdepartementet,?Akersgata 40, Postboks 8008 Dep, 0030 Oslo. http://www.regjeringen.no/upload/kilde/fin/reg/2005/0029/ddd/pdfv/266324-veileder_i_samfunnsok_analyse_trykket.pdf

[B13] Buechel FF Sr Long-term followup after mobile-bearing total knee replacement Clin Orthop Relat Res 2002 404 40 50 12439236 10.1097/00003086-200211000-00008

[B14] Gill GS Joshi AB Mills DM Total condylar knee arthroplasty. 16- to 21-year results Clin Orthop Relat Res 1999 367 210 215 10546617

[B15] Lidgren L Knutson K Robertsson O The Swedish knee arthroplasty register Annual report 2004 2004 4

[B16] Ranawat CS Flynn WF Jr Saddler S Hansraj KK Maynard MJ Long-term results of the total condylar knee arthroplasty. A 15-year survivorship study Clin Orthop Relat Res 1993 286 94 102 8425373

[B17] Furnes O Espehaug B Lie SA Vollset SE Engesaeter LB Havelin LI Failure mechanisms after unicompartmental and tricompartmental primary knee replacement with cement J Bone Joint Surg Am 2007 89 3 519 525 10.2106/JBJS.F.00210 17332100

[B18] Havelin LI Engesaeter LB Espehaug B Furnes O Lie SA Vollset SE The Norwegian Arthroplasty Register: 11 years and 73.000 arthroplasties Acta Orthop Scand 2000 71 4 337 353 10.1080/000164700317393321 11028881

[B19] Espehaug B Furnes O Havelin LI Engesaeter LB Vollset SE Kindseth O Registration completeness in the Norwegian Arthroplasty Register Acta Orthop 2006 77 1 49 56 10.1080/17453670610045696 16534702

[B20] Life tables Norway 2005, Statistisk sentralbyrå, Statistics Norway P.O. Box 8131 Dep.,NO-0033,Oslo,NORWAY**.** http://www.ssb.no/a/english/kortnavn/dode_en/arkiv/tab-2006-04-27-05-en.html

[B21] Mahomed NN Barrett J Katz JN Baron JA Wright J Losina E Epidemiology of total knee replacement in the United States Medicare population J Bone Joint Surg Am 2005 87 6 1222 1228 10.2106/JBJS.D.02546 15930530

[B22] Heck DA Robinson RL Partridge CM Lubitz RM Freund DA Patient outcomes after knee replacement Clin Orthop Relat Res 1998 356 93 110 9917673 10.1097/00003086-199811000-00015

[B23] Karrholm J Garellick G Rogmark C Herberts P Swedish hip arthroplasty register Annual report 2007 2007 67 69

[B24] Fryback DG Dasbach EJ Klein R Klein BE Dorn N Peterson K The Beaver Dam health outcomes study; initial catalog of health-state factors Med Decis Making 1993 13 89 102 10.1177/0272989X9301300202 8483408

[B25] Hunink MG Glasziou PP Siegel JE Weeks JC Pliskin JS Elstein AS Decision making in health and medicine 2001 The Edinburgh Building, Shaftesbury Road, Cambridge CB2 8RU, UK: Cambridge university press

[B26] Anderson KC Buehler KC Markel DC Computer assisted navigation in total knee arthroplasty: comparison with conventional methods J Arthroplasty 2005 20 7 Suppl 3 132 138 16214014 10.1016/j.arth.2005.05.009

[B27] Bolognesi M Hofmann A Computer navigation versus standard instrumentation for TKA: a single-surgeon experience Clin Orthop Relat Res 2005 440 162 169 10.1097/01.blo.0000186561.70566.95 16239801

[B28] Informasjonshefte Innsatsstyrt finansiering 2006 Helse- og omsorgsdepartementet 2006 Public information, ISBN: I-3/2006NB. Fagbokforlaget, Kanalveien 51, 5068 Bergen, Norway.

[B29] Guide to the methods of technology appraisal National Health Services, National Institute for Health and Clinical Excellence 2008 Paragraph 5.2.10. http://www.nice.org.uk N1618 1P June 2008, ISBN: 1-84629-741-9 27905712

[B30] Slover JD Tosteson AN Bozic KJ Rubash HE Malchau H Impact of hospital volume on the economic value of computer navigation for total knee replacement J Bone Joint Surg Am 2008 90 7 1492 1500 10.2106/JBJS.G.00888 18594098 PMC2657305

[B31] Furnes O Havelin LI Espehaug B Engesaeter LB Lie SA Vollset SE Det norske leddregisteret - 15 nyttige år for pasientene and for helsevesenet Tidsskr Nor Legeforen 2003 123 1367 12806680

[B32] Gothesen O Espehaug B Petursson G Havelin LI Furnes O Short term outcome of 1465 computer navigated primary total knee replacements. A report from the Norwegian Arthroplasty Register Acta Orthop Scand 2011 82 3 293 300 10.3109/17453674.2011.575743 PMC3235306 21504309

[B33] Arnesen T Trommald M Are QALYs based on time trade-off comparable?–A systematic review of TTO methodologies Health Econ 2005 14 1 39 53 10.1002/hec.895 15386674

[B34] Smabrekke A Espehaug B Havelin LI Furnes O Operating time and survival of primary total hip replacements: an analysis of 31.745 primary cemented and uncemented total hip replacements from local hospitals reported to the Norwegian Arthroplasty Register 1987–2001 Acta Orthop Scand 2004 75 5 524 532 10.1080/00016470410001376 15513482

