# Supplementary material for: An economic model to evaluate cost-effectiveness of computer assisted knee replacement surgery in Norway
Source: BMC Musculoskelet Disord. 2013 Jul 6;14:202. doi: 10.1186/1471-2474-14-202 (PMC3722089; doi:10.1186/1471-2474-14-202)
Supplement: Additional file 1 — Appendix. [file 1471-2474-14-202-S1.doc]

Appendix

If we disregard the NOK 500,000 threshold and purely look for the most cost saving (cost effective) alternative, the implant survival rate required to reduce the cost per QALY to the same level as for TKA (table, figure 2-5) will be considerably higher. We would then be assuming that CAS is better than TKA, but that there is no more money available to spend. Given this requirement, the implant survival in cohort 1 will need to increase from 89.8% to 90.6-95.7%, and in cohort 2 from 95.1% to 95.4-97.6%, depending on patient volume and the cost of the navigation equipment (tab 5 below). Again, the requirement for an improved implant survival rate is lowest at high patient volumes. Doubling the cost without a threshold will impact at both high and low patient volumes, with a requirement for further improvement of the 10-year implant survival rate. A somewhat larger reduction of the probability of revision is required in the younger chort (8.0-58.0%) than in the older cohort (5.6-50.0%) in order to achieve the cost reduction (cost effectiveness) required.


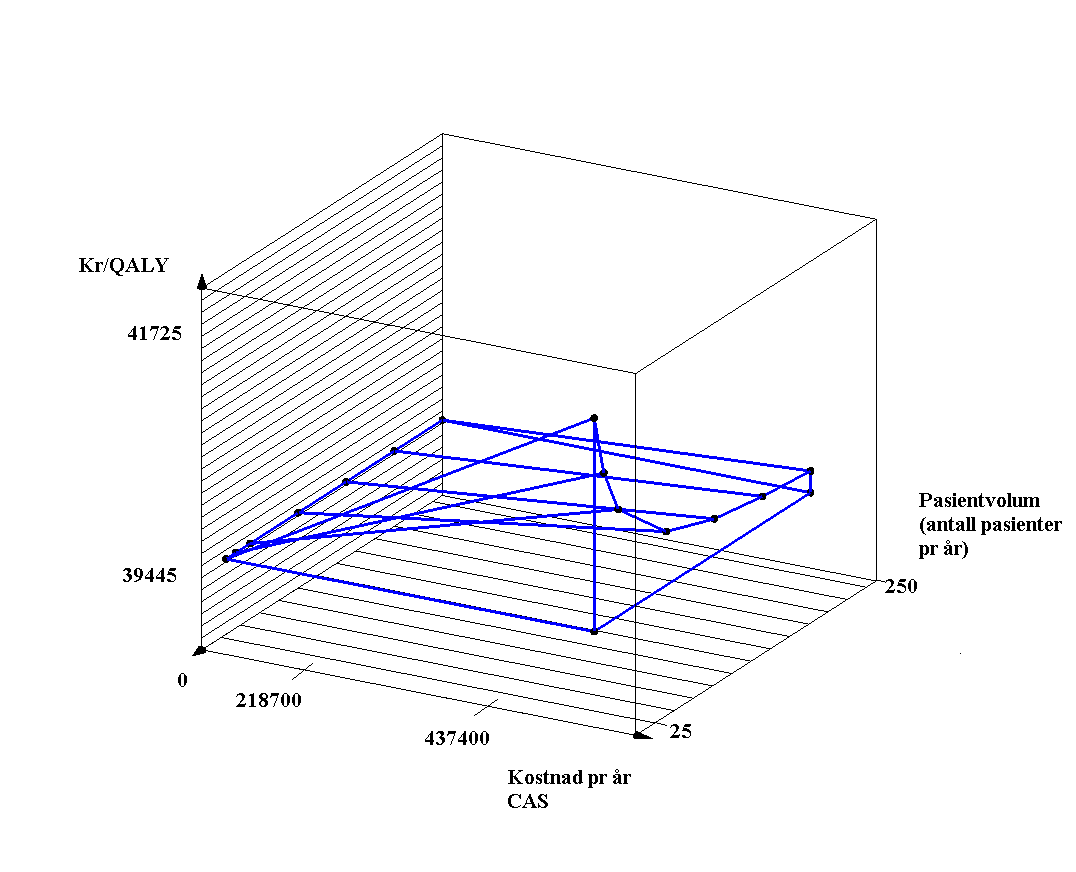


NOK / QUALY

Cost per year, CAS

Patient volume (number of patients per year)

Figure X. 3D diagram showing the connection between increased costs on the X axis, cost per QALY on the Y axis and patient volume on the Z axis for cohort 1.


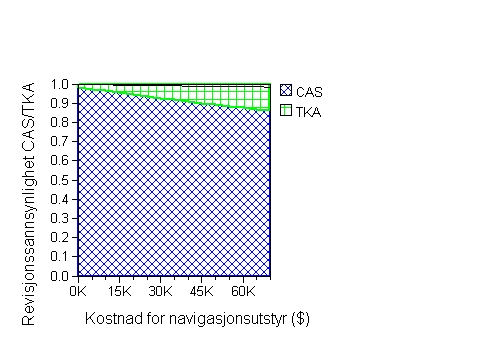


Probability of revision CAS/TKA

Cost of navigation equipment (USD)

Figure 5a (high patient volume, cohort 1, age 60)

We see that at our basic price of USD 33,960 (NOK 216,500), the probability of revision needs to be reduced by 1.0% for the threshold not to be exceeded, and by 8.0% to achieve cost effectiveness. If the costs are doubled the probability of revision needs to be reduced by 1.7% not to exceed the threshold, and 13.5% to achieve cost effectiveness.


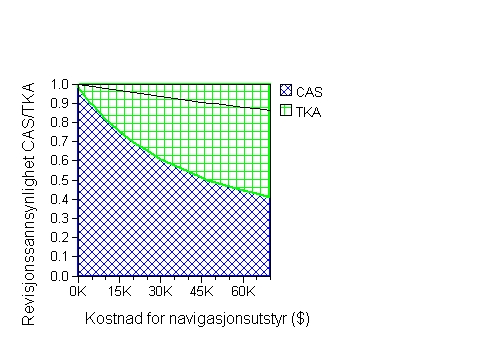


Probability of revision CAS/TKA

Cost of navigation equipment (USD)

Figure 5b (low patient volume, cohort 1, age 60).

We see that at our basic price of USD 33,960 (NOK 216,500) the probability of revision needs to be reduced by 7.5% not to exceed the threshold, and by 42.0% to achieve cost effectivenesss. If the costs are doubled, the probability of revision needs to be reduced by 13.0% not to exceed the threshold, and by 58.0% to achieve cost effectiveness.


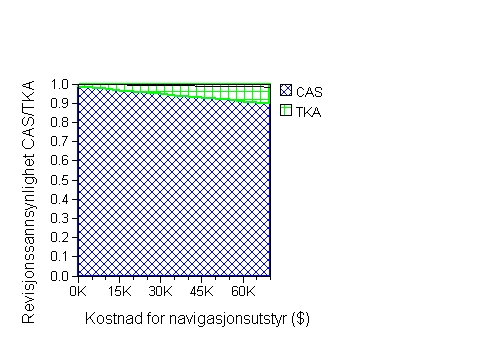


Probability of revision CAS/TKA

Cost of navigation equipment (USD)

Figure 6a (high patient volume, cohort 2, age 75)

Figures 6a and 6b show the impact of costs on cost effectiveness in the older cohort at high and low patient volumes respectively.


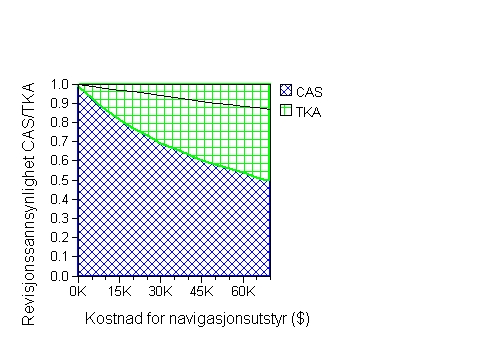


Probability of revision CAS/TKA

Cost of navigation equipment (USD)

Figure 6b (low patient volume, cohort 2, age 75)

|  | Yearly probability of revision | Implant survival  (Kaplan-Meier) | Probability of revision in the course of the first 10 years | Yearly probability of revision | Implant survival  (Kaplan-Meier) | Probability of revision in the course of 10 years |
| --- | --- | --- | --- | --- | --- | --- |
| Index year | <70 | <70 |  | >=70 | >=70 |  |
| 1 | 1.60 |  |  | 1.25 |  |  |
| 2 | 2.30 |  |  | 1.29 |  |  |
| 3 | 1.40 |  |  | 0.79 |  |  |
| 4 | 1.20 |  |  | 0.55 |  |  |
| 5 | 1.00 | 95.00% |  | 0.52 | 97.20% |  |
| 6 | 1.05 |  |  | 0.38 |  |  |
| 7 | 1.05 |  |  | 0.37 |  |  |
| 8 | 1.37 |  |  | 0.23 |  |  |
| 9 | 0.70 |  |  | 0.63 |  |  |
| 10 | 0.96 | 89.75% | 10.25 | 0.25 | 95.10% | 4.90 |
| 11 | 1.02 |  |  | 0.50 |  |  |
| 12 | 0.50 |  |  | 0.50 |  |  |
| 13 | 0.50 |  |  | 0.50 |  |  |
| 14 | 0.50 |  |  | 0.50 |  |  |
| 15 | 0.50 | 86.75% |  | 0.50 | 90.10% |  |
| 16 | 1.00 |  |  | 0.50 |  |  |
| 17 | 1.00 |  |  | 0.50 |  |  |
| 18 | 1.00 |  |  | 0.50 |  |  |
| 19 | 1.00 |  |  | 0.50 |  |  |
| 20 | 1.00 | 81.75% |  | 0.50 | 85.50% |  |

Table A. Yearly probability of revision in the two cohorts. Kaplan-Meier implant survival at 5, 10, 15 and 20 years.

|  |  |  |  |  |  |  |  |
| --- | --- | --- | --- | --- | --- | --- | --- |
| 60 | 0.010152 | 70 | 0.023668 | 80 | 0.057067 | 90 | 0.143407 |
| 61 | 0.010869 | 71 | 0.025812 | 81 | 0.065032 | 91 | 0.155088 |
| 62 | 0.012066 | 72 | 0.028285 | 82 | 0.067581 | 92 | 0.167323 |
| 63 | 0.013155 | 73 | 0.031088 | 83 | 0.077566 | 93 | 0.180093 |
| 64 | 0.014333 | 74 | 0.033548 | 84 | 0.085272 | 94 | 0.193378 |
| 65 | 0.015584 | 75 | 0.036749 | 85 | 0.093544 | 95 | 0.207148 |
| 66 | 0.016855 | 76 | 0.040177 | 86 | 0.102375 | 96 | 0.221372 |
| 67 | 0.018535 | 77 | 0.043642 | 87 | 0.111774 | 97 | 0.236010 |
| 68 | 0.020125 | 78 | 0.048232 | 88 | 0.121744 | 98 | 0.251018 |
| 69 | 0.021928 | 79 | 0.052885 | 89 | 0.132290 | 99 | 0.266346 |
|  |  |  |  |  |  | 100 | 1 |

Table B. Age-specific death rates. Mortality table 2005, males and females. (Statistics Norway)

| Table C. Overview of probabilities of revision and impant survival rates that produce cost effectiveness or that represent the limit for what the healthcare sector is willing to pay (threshold), given the specified prices, patient volumes and age cohorts. (Compared with the current 10-year implant survival rate for 60-year-olds of 89.8% and for 75-year-olds of 95.1%) | | | | | | | | | | |
| --- | --- | --- | --- | --- | --- | --- | --- | --- | --- | --- |
|  |  | Cost effective | | | |  | Values indicating the healthcare sector’s threshold ^(3)^ for willingness to pay | | | |
| Cohort 1 | | High volume^(1)^ Price 1^(4)^ | Low volume^(2)^  Price 1 | High volume  Price 2^(5)^ | Low volume  Price 2 |  | High volume  Price 1 | Low volume  Price 1 | High volume  Price 2 | Low volume  Price 2 |
|  | Reduction in probability of revision (%) | 8.0 | 42.0 | 13.5 | 58.0 |  | 1.0 | 7.5 | 1.7 | 13.0 |
|  | New 10-year implant survival rate  (%) | 90.6 | 94.1 | 91.2 | 95.7 |  | 89.9 | 90.6 | 89.9 | 91.1 |
|  |  |  |  |  |  |  |  |  |  |  |
| Cohort 2 | | High volume  Price 1 | Low volume  Price 1 | High volume  Price 2 | Low volume  Price 2 |  | High volume  Price 1 | Low volume  Price 1 | High volume Price 2 | Low volume  Price 2 |
|  | Reduction in probability of revision  (%) | 5.6 | 33.0 | 10.0 | 50.0 |  | 0.8 | 7.0 | 1.5 | 12.7 |
|  | New 10-year implant survival rate  (%) | 95.4 | 96.7 | 95.6 | 97.6 |  | 95.14 | 95.4 | 95.2 | 95.7 |
|  |  |  |  | | | | | | |  |
| ^(1)^250 knee prostheses per year, ^(2)^25 knee prostheses per year, ^(3)^NOK 500,000 per QALY, ^(4)^NOK 216,500, ^(5)^NOK 433,000 | | | | | | | | | | |

| Table D. Overview of the connection between changes to utility values following a change in the probability of revision, as well as the impact of age (cohorts 1 and 2) and patient volume on cost effectiveness and the probability of not exceeding the ICER threshold of NOK 500,000. | | | | | | |
| --- | --- | --- | --- | --- | --- | --- |
|  | Measure of benefit Δ QALY (QALY CAS – QALY TKA) | | Cost added and (ICER) per computer assisted knee replacement, given in NOK (cost CAS – cost TKA) | | | |
|  | Cohort 1  (60-year-olds) | Cohort 2  (75-year-olds) | Cohort 1 (60-year-olds) |  | Cohort 2 (75-year-olds) |  |
| Reduction in probability of revision (%) |  |  | Low volume (25) | High volume (250) | Low volume (25) | High volume (250) |
| 0 | 0 | 0 | 7718 | 951 | 7718 | 951 |
| 1 | 0.001757 | 0.001760 | 7609  (4330677) | **843**  **(479795)** | 7562  (4296591) | **795**  **(451705)** |
| 2 | 0.003547 | 0.003552 | 7499  (2114180) | 733  (206654) | 7403  (2084178) | 636  (179054) |
| 3 | 0.005372 | 0.005377 | 7386  (1374907) | 619  (115227) | 7240  (1346476) | 474  (88153) |
| 4 | 0.007232 | 0.007237 | 7271  (1005393) | 505  (69829) | 7075  (977615) | 309  (42697) |
| 5 | 0.009129 | 0.009133 | 7143  (782452) | 388  (42502) | 6906  (756159) | 139  (15220) |
| 6 | 0.011063 | 0.011065 | 7034  (635813) | 268  (24225) | 6734  (608586) | **-33**  **(-2982)** |
| 7 | 0.013036 | 0.013035 | 6912  (530224) | 146  (11200) | 6558  (503107) | -209  (-16034) |
| 8 | 0.015050 | 0.015043 | **6788**  **(451030)** | 21  (1395) | **6378**  **(423985)** | -388  (-25793) |
| 9 | 0.017104 | 0.017092 | 6661  (389441) | **-106**  **(-6197)** | 6195  (362450) | -571  (-33407) |
| 10 | 0.019202 | 0.019183 | 6531  (340121) | -236  (-12290) | 6008  (313194) | -758  (-39514) |
|  |  |  |  |  |  |  |
| 20 | 0.042886 | 0.042693 | 5060  (117987) | -1707  (-39803) | 3882  (90928) | -2885  (-67575) |
|  |  |  |  |  |  |  |
| 30 | 0.072985 | 0.072300 | 3177  (43529) | -3590  (-49188) | 1151  (15920) | -5615  (-77663) |
| 32 | 0.080026 | 0.079177 | 2734  (34164) | -4033  (-50396) | 507  (6403) | -6259  (-79051) |
| 33 | 0.083699 | 0.082757 | 2501  (29881) | -4265  (-50956) | 171  (2066) | -6596  (-79703) |
| 34 | 0.087479 | 0.086436 | 2264  (25880) | -4503  (-51475) | **-176**  **(-2036)** | -6943  (-80325) |
| 36 | 0.095384 | 0.094110 | 1763  (18483) | -5004  (-52462) | -904  (-9606) | -7671  (-81511) |
| 38 | 0.103784 | 0.102236 | 1230  (11852) | -5536  (-53342) | -302  (-15777) | -8247  (-80666) |
| 40 | 0.112728 | 0.110856 | 661  (5864) | -6105  (-54157) | -2509  (-22633) | -9276  (-83676) |
| 42 | 0.122271 | 0.120015 | 55  (450) | -6715  (-54919) | -3398  (-28313) | -10164  (-84689) |
| 43 | 0.127287 | 0.124812 | **-270**  **(-2121)** | -7037  (-55285) | -3866  (-30975) | -10633  (-85189) |
| 44 | 0.132477 | 0.129764 | -604  (-4559) | -7371  (-55640) | -4351  (-33530) | -11118  (-85679) |
| 46 | 0.143417 | 0.140161 | -1310  (-9134) | -8077  (-56318) | -5377  (-38363) | -12144  (-86643) |
| 48 | 0.155171 | 0.151271 | -2073  (-13359) | -8840  (-56969) | -6486  (-42877) | -13253  (-87611) |
| 50 | 0.167834 | 0.163167 | -2901  (-17285) | -9667  (-57599) | -7686  (-47105) | -14452  (-88572) |
|  |  |  |  |  |  |  |
| 60 | 0.249111 | 0.237708 | -8387  (-33668) | -15154  (-60833) | -15506  (-65231) | -22273  (-93699) |
|  |  |  |  |  |  |  |
| 90 | 1.017135 | 0.837304 | -80034  (-78686) | -86801  (-85339) | -94317  (-112643) | -101084  (-120726) |
